# Supplementary material for: Return of naturally sourced Pb to Atlantic surface waters
Source: Nat Commun. 2016 Sep 28;7:12921. doi: 10.1038/ncomms12921 (PMC5052796; doi:10.1038/ncomms12921)
Supplement: Supplementary Information — Supplementary Figures 1-6, Supplementary Tables 1-8, Supplementary Notes 1-2 and Supplementary References. [file ncomms12921-s1.pdf]

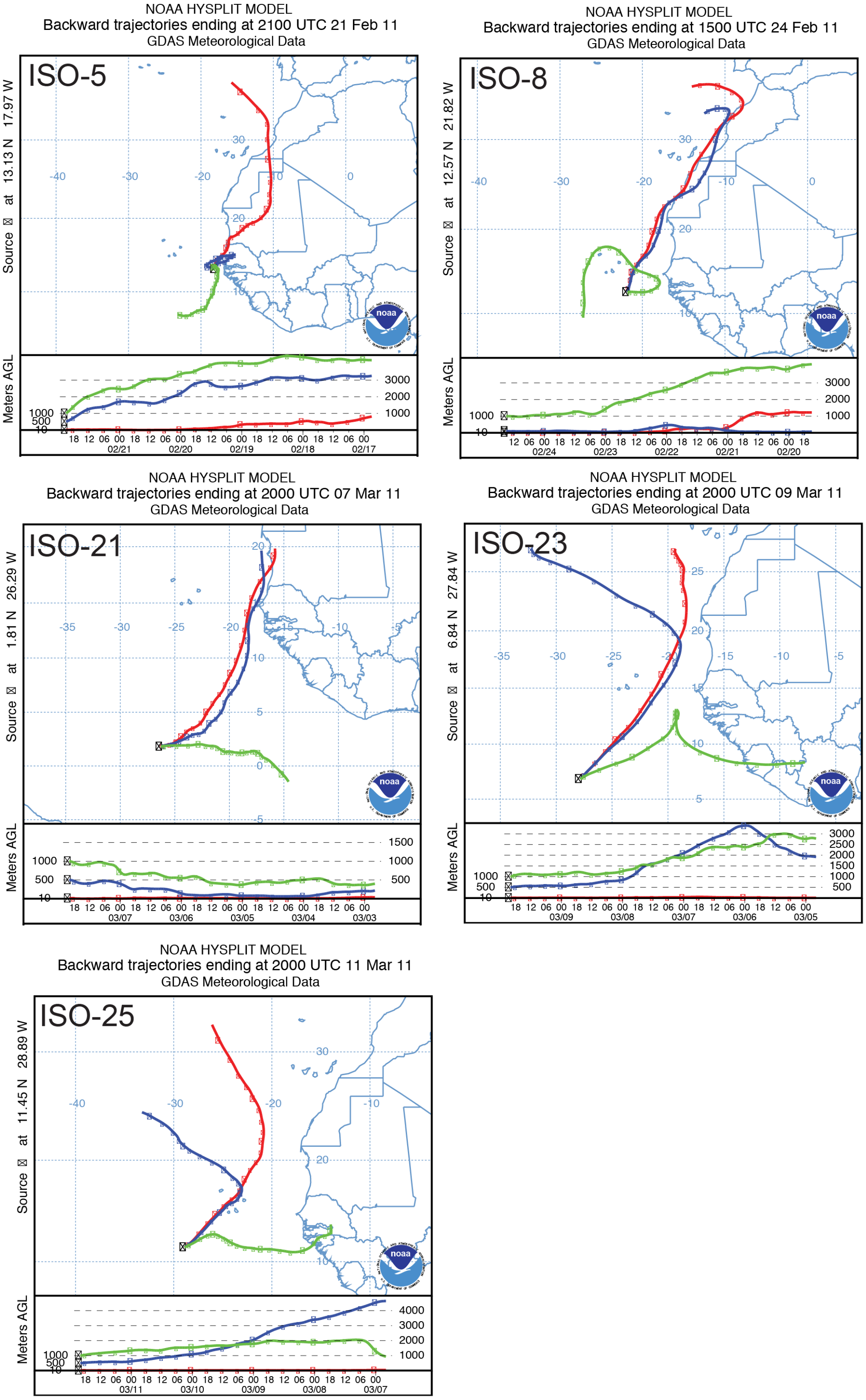

Supplementary Figure 1; Air mass back trajectories for the 5 days preceding the start of each collection of aerosols of the North African group. Calculated using the NOAA Air Resources Laboratory Hybrid Single-Particle Lagrangian Integrated Trajectory Model, (HYSPLIT, GDAS data set)[25]. Arrival heights of 10, 500 and 1000

m were used along with model vertical velocity for calculation of vertical motion. AGL= above ground level.

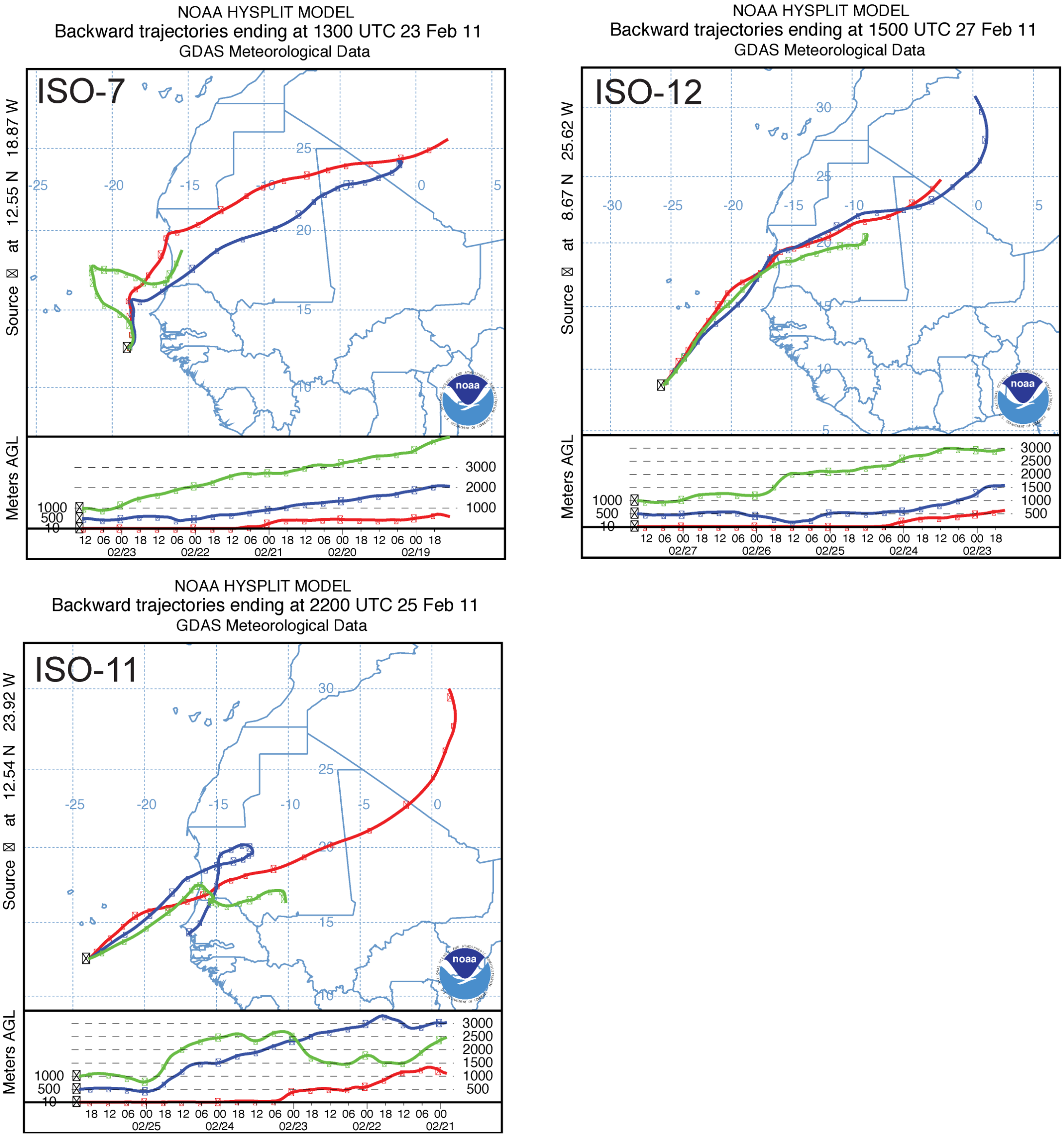

Supplementary Figure 2; Air mass back trajectories for the 5 days preceding the start of each collection of aerosols of the Algerian group. Calculated using the NOAA Air Resources Laboratory Hybrid Single-Particle Lagrangian Integrated Trajectory Model, (HYSPLIT, GDAS data set)[25]. Arrival heights of 10, 500 and 1000 m were used along with model vertical velocity for calculation of vertical motion. AGL= above ground level

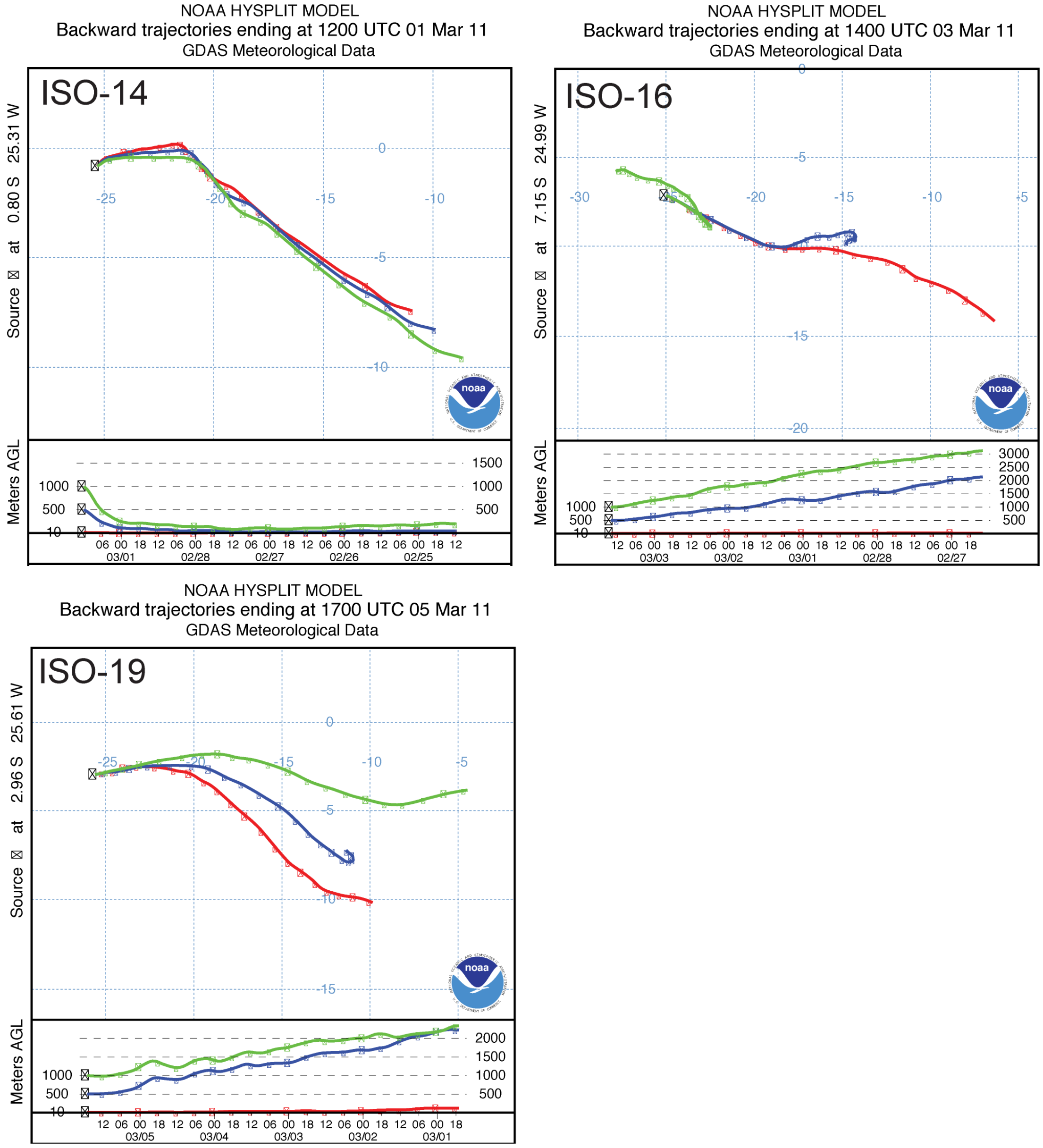

Supplementary Figure 3; Air mass back trajectories for the 5 days preceding the start of each collection of aerosols of the Oceanic group. Calculated using the NOAA Air Resources Laboratory Hybrid Single-Particle Lagrangian Integrated Trajectory Model, (HYSPLIT, GDAS data set)[25]. Arrival heights of 10, 500 and 1000 m were used along with model vertical velocity for calculation of vertical motion. AGL=above ground level

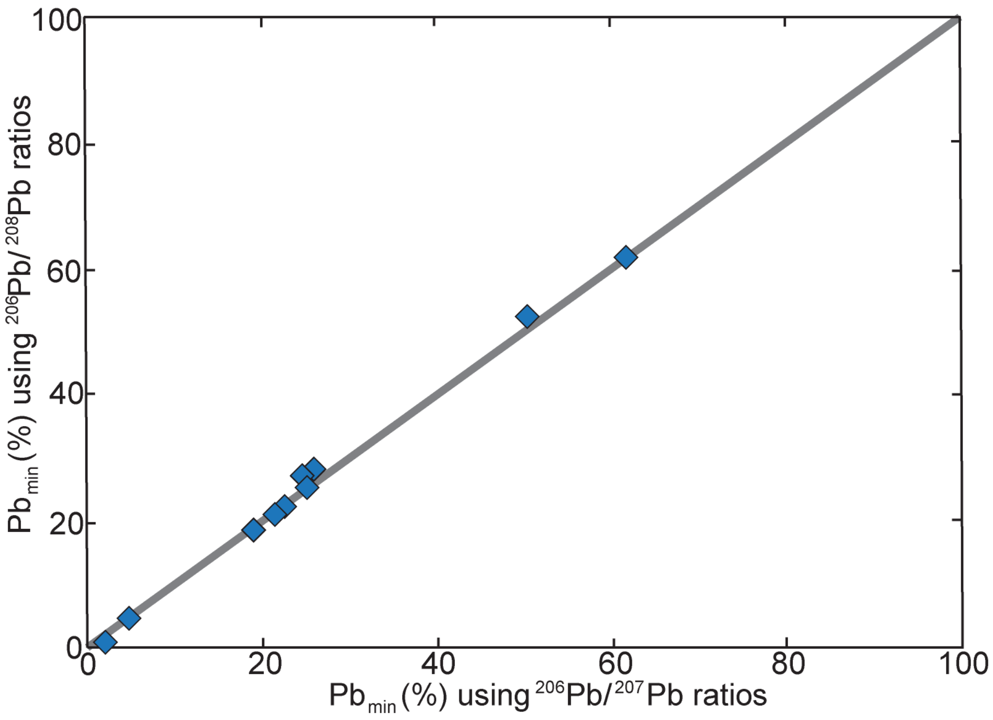

Supplementary Figure 4; Estimates on the contribution of Pb from mineral dust in aerosol total digests using mass balance considerations based on $^{206}Pb/^{207}Pb$ and $^{208}Pb/^{207}Pb$ respectively. The grey line denotes a 1:1 relationship. Pb_{min} = mineral dust derived Pb.

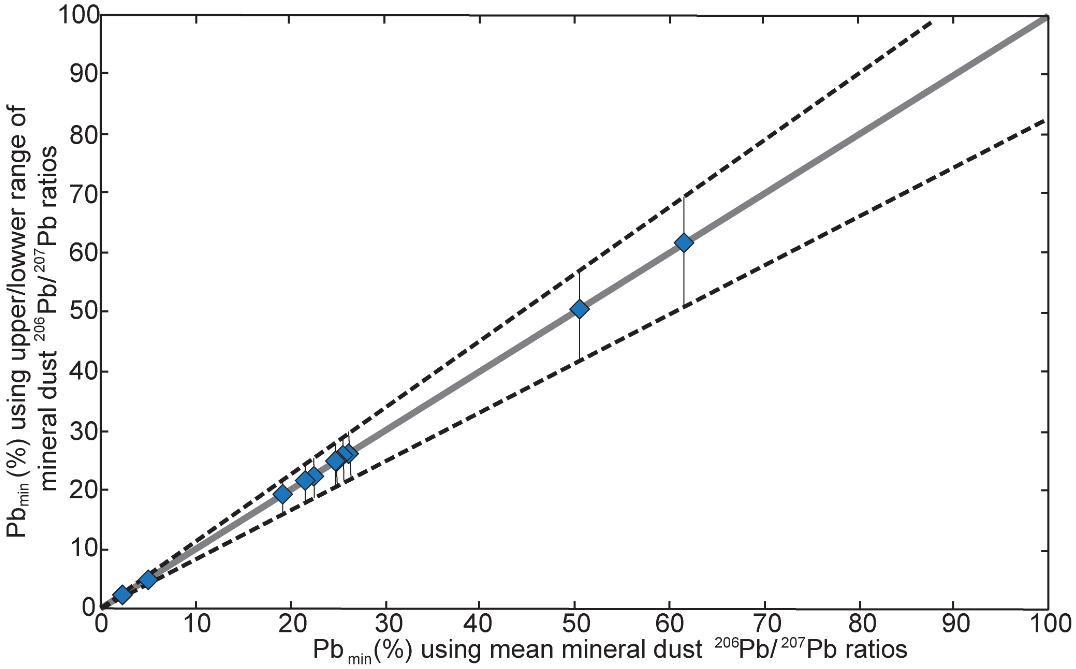

Supplementary Figure 5; Uncertainty in the estimates on the contribution of Pb from mineral dust in aerosol total digests using the isotope mass balance approach. The uncertainty in these estimates is calculated by propagating the full range of $^{206}Pb/^{207}Pb$ ratios of the compiled mineral dust literature data through the calculation as denoted by the dashed lines. Pb_{min} = mineral dust derived Pb.

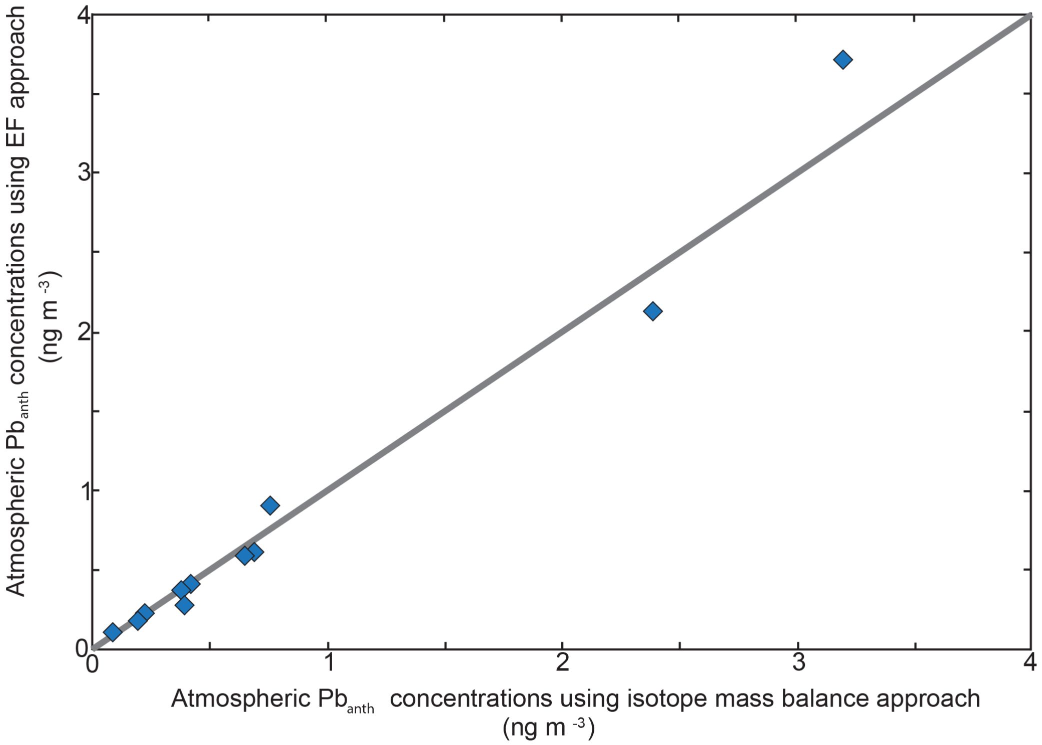

Supplementary Figure 6; A comparison of the calculated atmospheric anthropogenic Pb concentrations based on two independent approaches to derive mineral dust Pb proportions: the isotope mass balance approach and enrichment factor (EF) approach. The grey line denotes a 1:1 relationship. Pb$_{anth}$ = anthropogenic derived Pb.

Supplementary Table 1; Assessment of particulate Pb contributions to unfiltered samples by comparing Pb concentrations for unfiltered, filtered (<0.2 µm), and particulate samples (>0.45 µm).

Sample ID	Latitude (°N)	Longitude (°W)	Depth (m)	Type	Pb concentration (pmol kg^{-1})	Particulate Pb contribution (%)
GEOTRACES section GA02, leg 2, PE321 Western Tropical Atlantic (WTA), 11th June – 8th July, 2010						
FISH 13b	31.70	64.24	2-3	Unfiltered	21.5	-0.3%
Stn 21	31.67	64.17	9	Filtered	21.6	
FISH 13a	29.70	66.44	2-3	Unfiltered	22.5	-6.2%
Stn 22	29.62	67.53	25	Filtered	23.9	
FISH 14	28.04	67.51	2-3	Unfiltered	22.3	-37.1%
Stn 23	28.09	67.50	11	Filtered	30.5*	
FISH 16a	26.06	67.72	2-3	Unfiltered	24.2	5.4%
Stn 24	26.24	67.80	10	Filtered	22.9	
FISH 16b	24.75	67.10	2-3	Unfiltered	23.3	-1.2%
Stn 25	24.71	67.07	10	Filtered	23.6	
FISH 17	23.30	65.58	2-3	Unfiltered	22.1	15.5%
Stn 26	23.27	65.55	11	Filtered	18.7	
FISH 18	22.38	63.67	2-3	Unfiltered	23.2	5.1%
Stn 27	22.34	63.58	12	Filtered	22.0	
FISH 19	21.79	61.89	2-3	Unfiltered	21.9	-7.5%
Stn 28	21.78	61.84	12	Filtered	23.5	
FISH 20	20.56	59.72	2-3	Unfiltered	23.0	8.3%
Stn 29	20.46	59.53	10	Filtered	21.1	
FISH 21	18.74	57.77	2-3	Unfiltered	19.1	7.1%
Stn 30	18.57	57.61	11	Filtered	17.8	
FISH 22	16.77	56.22	2-3	Unfiltered	16.9	17.9%
Stn 31	16.83	56.27	9	Filtered	13.9	
FISH 23	14.80	54.74	2-3	Unfiltered	16.8	20.9%
Stn 32	14.88	54.80	10	Filtered	13.3	
FISH 24	13.15	53.41	2-3	Unfiltered	15.0	33.7%
Stn 33	13.16	53.42	10	Filtered	9.9	
FISH 25	11.52	52.15	2-3	Unfiltered	17.3	-1.5%
Stn 34	11.37	52.05	8	Filtered	17.6	
FISH 26	9.50	50.43	2-3	Unfiltered	19.8	11.0%
Stn 35	9.55	50.47	10	Filtered	17.6	
FISH 27	7.73	48.84	2-3	Unfiltered	20.8	3.0%
Stn 36	7.77	48.88	10	Filtered	20.2	
FISH 28	5.93	46.36	2-3	Unfiltered	17.7	3.8%
Stn 37	5.98	46.42	9	Filtered	17.1	
FISH 29	4.19	44.04	2-3	Unfiltered	18.1	-2.4%
Stn 38	3.98	43.75	9	Filtered	18.6	
FISH 30	2.62	41.81	2-3	Unfiltered	19.2	12.0%
Stn 39	2.54	41.70	9	Filtered	16.9	
FISH 31	1.19	39.74	2-3	Unfiltered	19.6	5.7%
Stn 40	1.15	39.69	10	Filtered	18.5	
FISH 32	0.70	38.97	2-3	Unfiltered	23.9	16.3%
Stn 41	0.72	38.97	11	Filtered	20.0	

Supplementary Table 1; continued.

Sample ID	Latitude (°N)	Longitude (°W)	Depth (m)	Type	Pb concentration (pmol kg-1)	Particulate Pb contribution (%)
GEOTRACES section GA06, D361, Eastern Tropical Atlantic (ETA), 7th February – 19th March, 2011						
FISH 43	12.59	17.65	2-3	Unfiltered	11.5	45.6%
Stn 2	12.35	17.55	24	Particulate	5.260	
FISH 164	-3.31	25.49	2-3	Unfiltered	20.4	7.2%
Stn 12	-1.10	25.47	24	Particulate	1.476	
FISH 200	8.28	28.31	2-3	Unfiltered	15.9	11.9%
Stn 16	8.20	28.20	25	Particulate	1.893	
FISH 211	11.90	28.92	2-3	Unfiltered	18.8	8.0%
Stn 18	12.00	29.00	24	Particulate	1.500	
FISH 227	17.37	28.39	2-3	Unfiltered	16.5	8.0%
Stn 20	17.23	28.23	24	Particulate	1.318	

*Sample is suspected of contamination.

Lead concentrations of filtered samples were converted from pmol l^{-1} to pmol kg^{-1} assuming a water density of 1025kg m^{-3}. Note relative uncertainty of the unfiltered Pb concentrations is approximately ±5%, therefore particulate Pb contributions are subject to similar levels of uncertainty.

Supplementary Table 2; Compiled literature data used to assess the isotope composition of anthropogenic Pb transported by the easterly winds.

Location	Year	Leaching protocols	$^{206}Pb/^{207}Pb$	$^{208}Pb/^{207}Pb$	Reference
Tunisia	1995	0.4 HBr (1 hr)	1.1430	2.4200	2
Morocco	1997	0.4 HBr (1 hr)	1.1410	2.4180	2
Egypt	1995	0.4 HBr (1 hr)	1.1530	2.4310	2
Senegal	1995	0.4 HBr (1 hr)	1.1010	2.3750	2
Mail	2008	0.5 HBr (15 mins)	1.1536*	2.4349*	3
Mali	2008	0.5 HBr (15 mins)	1.1698*	2.4530*	3
Mali	2008	0.5 HBr (15 mins)	1.1590*	2.4418*	3
Mali	2008	0.5 HBr (15 mins)	1.1596*	2.4408*	3
Cape Verde	2008	0.5 HBr (15 mins)	1.1235*	2.4056*	3
Tropical Atlantic	2001	0.1 M HNO_3 (24 hr)	1.1450	2.4180	1
Tropical Atlantic	2001	0.1 M HNO_3 (24 hr)	1.1600	2.4350	1
Tropical Atlantic	2001	0.1 M HNO_3 (24 hr)	1.1650	2.4240	1
Tropical Atlantic	2001	0.1 M HNO_3 (24 hr)	1.1560	2.4300	1
Tropical Atlantic	2001	0.1 M HNO_3 (24 hr)	1.1540	2.4300	1
Tropical Atlantic	2001	0.1 M HNO_3 (24 hr)	1.1660	2.4540	1
Tropical Atlantic	2001	0.1 M HNO_3 (24 hr)	1.1630	2.4380	1
Tropical Atlantic	2001	0.1 M HNO_3 (24 hr)	1.1490	2.4230	1
Tropical Atlantic	2011	AA (pH 4.7) (1 hr)	1.1668	2.4462	This study (ISO-5_{leach})
Tropical Atlantic	2011	AA (pH 4.7) (1 hr)	1.1587	2.4378	This study (ISO-7_{leach})
Tropical Atlantic	2011	AA (pH 4.7) (1 hr)	1.1536	2.4314	This study (ISO-8_{leach})
Tropical Atlantic	2011	AA (pH 4.7) (1 hr)	1.1567	2.4331	This study (ISO-11_{leach})
Tropical Atlantic	2011	AA (pH 4.7) (1 hr)	1.1597	2.4360	This study (ISO-12_{leach})
Tropical Atlantic	2011	AA (pH 4.7) (1 hr)	1.1652	2.4438	This study (ISO-21_{leach})
Tropical Atlantic	2011	AA (pH 4.7) (1 hr)	1.1547	2.4292	This study (ISO-23_{leach})
Tropical Atlantic	2011	AA (pH 4.7) (1 hr)	1.1534	2.4298	This study (ISO-25_{leach})

AA - ammonium acetate.

* Pb isotope ratios quoted in the table ($^{206}Pb/^{207}Pb$ and $^{208}Pb/^{207}Pb$) were calculated from $^{206}Pb/^{204}Pb$, $^{207}Pb/^{204}Pb$ and $^{208}Pb/^{204}Pb$ ratios quoted in the original publication.

Supplementary Table 3; Compiled literature data used to assess the isotope composition of anthropogenic Pb transported by the westerly winds.

Location	Year	Leaching protocols	$^{206}Pb/^{207}Pb$	$^{208}Pb/^{207}Pb$	Reference
Argonne (US)	1995	0.4 M HBr (1 hr)	1.2110	2.4570	2
Wilmington (US)	1995	0.4 M HBr (1 hr)	1.2330	2.4610	2
South Holland (US)	1995	0.4 M HBr (1 hr)	1.2200	2.4590	2
Sioux (US)	1996	0.4 M HBr (1 hr)	1.1950	2.4500	2
Dallas (US)	1997	0.4 M HBr (1 hr)	1.2020	2.4450	2
New York (US)	1998	0.4 M HBr (1 hr)	1.1970	2.4590	2
Tampa (US)	1994	0.4 M HBr (1 hr)	1.2140	2.4520	2
Tampa (US)	1998	0.4 M HBr (1 hr)	1.2080	2.4590	2
Woods Hole (US)	1998	0.4 M HBr (1 hr)	1.1730	2.4380	2
Havanna	1997	0.4 M HBr (1 hr)	1.1610	2.4380	2
Mexico City	1998	0.4 M HBr (1 hr)	1.1900	2.4600	2
Mexico City	1998	0.4 M HBr (1 hr)	1.1880	2.4520	2
Santa Ana	1994	0.4 M HBr (1 hr)	1.2150	2.4600	2
Puerto Rico	1994	0.4 M HBr (1 hr)	1.1760	2.4290	2
Cuba-Habana	1995	0.4 M HBr (1 hr)	1.1610	2.4380	2
North Atlantic	2011	Deionized H_2O*	1.1674	2.4262	5
North Atlantic	2011	Deionized H_2O*	1.1712	2.4346	5
North Atlantic	2011	Deionized H_2O*	1.1647	2.4321	5

*Aerosol samples were leached following the methods of Buck et al.[6]

Supplementary Table 4; Compiled literature data used to assess the Pb isotope composition of suspended riverine particles in the Amazon basin.

River	$^{206}Pb/^{207}Pb$	$^{208}Pb/^{207}Pb$	Reference
Negro	1.2071	2.4715	8
Solimoes	1.2077	2.4785	8
Amazon	1.2062	2.4788	8
Madeira	1.1923	2.4804	8
Amazon	1.2089	2.4829	8
Urucara	1.2131	2.4780	8
Trombetas	1.2528	2.5075	8
Tapajos	1.2093	2.4825	8
Amazon	1.2058	2.4849	8
Amazon	1.2024	2.4957	7

All Pb isotope ratios quoted in the table ($^{206}Pb/^{207}Pb$ and $^{208}Pb/^{207}Pb$) were calculated from $^{206}Pb/^{204}Pb$, $^{207}Pb/^{204}Pb$ and $^{208}Pb/^{204}Pb$ ratios provided in the original publication.

Supplementary Table 5; Compiled literature data used to assess the Pb isotope composition of North African mineral dust.

Type	Location	$^{206}Pb/^{207}Pb$	$^{208}Pb/^{207}Pb$	Reference
PMS	Sierra Leone Rise	1.2051	2.4918	10
PMS	Sierra Leone Rise	1.2048	2.4916	10
PMS	Sierra Leone Rise	1.2039	2.4930	10
PMS	Sierra Leone Rise	1.2035	2.4905	10
PMS	Sierra Leone Rise	1.2025	2.4905	10
PMS	Sierra Leone Rise	1.2029	2.4906	10
PMS	Sierra Leone Rise	1.2029	2.4931	10
PMS	Sierra Leone Rise	1.2027	2.4919	10
PMS	Sierra Leone Rise	1.2028	2.4923	10
PMS	Sierra Leone Rise	1.2021	2.4918	10
PMS	Sierra Leone Rise	1.2022	2.4918	10
PMS	Sierra Leone Rise	1.2028	2.4918	10
PMS	Sierra Leone Rise	1.2055	2.4924	10
PMS	Sierra Leone Rise	1.2027	2.4883	10
PMS	Sierra Leone Rise	1.2019	2.4915	10
PMS	Sierra Leone Rise	1.2026	2.4924	10
PMS	Sierra Leone Rise	1.2024	2.4914	10
PMS	ETA	1.2000	2.5000	9
PMS	ETA	1.2010	2.5021	9
PMS	ETA	1.2030	2.5010	9
PMS	ETA	1.2030	2.4959	9
PMS	ETA	1.2020	2.4990	9
N.A dust	Bodélé depression	1.2112	2.5076	11
N.A dust	Bodélé depression	1.2046	2.4966	11
N.A dust	Bodélé depression	1.2037	2.4977	11
N.A dust	Bodélé depression	1.2115	2.5134	11
N.A dust	Bodélé depression	1.2167	2.5117	11
N.A dust	Bodélé depression	1.2153	2.5105	11
N.A dust	Bodélé depression	1.2100	2.5138	11
N.A dust	Bodélé depression	1.2161	2.5105	11

PMS = Pleistocene marine sediment, N.A dust = North African dust collected in source region (Bodélé depression, Chad), ETA = Eastern Tropical Atlantic.

The $^{206}Pb/^{207}Pb$ and $^{208}Pb/^{207}Pb$ ratios quoted in the table were calculated from $^{206}Pb/^{204}Pb$, $^{207}Pb/^{204}Pb$ and $^{208}Pb/^{204}Pb$ ratios quoted in [8,9] and from $^{206}Pb/^{207}Pb$ and $^{208}Pb/^{207}Pb$ quoted in ref[7].

Supplementary Table 6; Metadata for the aerosol samples collected for isotopic analysis during the GEOTRACES GA06 section cruise D361.

**Sample** [a]	**Starting Latitude (°N)**[b]	**Ending Latitude (°N)**[b]	**Starting Longitude (°W)**[b]	**Ending Longitude (°W)**[b]	**Start date/ time**[c]	**End date/ time**[c]	**Volume of air sampled (m^3)**[d]	**Proportion of exposed filter area (%)**[e]
ISO-5_{Total}<br>ISO-5_{Leach}	13.13	12.55	17.97	18.84	21/02/2011<br>21:24	23/02/2011<br>11:38	1981.9	12.5<br>37.5
ISO-7_{Total}<br>ISO-7_{Leach}	12.55	12.57	18.87	21.82	23/02/2011<br>13:10	24/02/2011<br>13:29	1462.2	50<br>50
ISO-8_{Total}<br>ISO-8_{Leach}	12.57	12.57	21.82	23.76	24/02/2011<br>14:49	25/02/2011<br>20:57	1813.8	50<br>50
ISO-11_{Total}<br>ISO-11_{Leach}	12.54	9.46	23.92	25.66	25/02/2011<br>22:00	27/02/2011<br>10:28	1006.2	50<br>50
ISO-12_{Total}<br>ISO-12_{Leach}	8.67	1.02	25.62	25.33	27/02/2011<br>14:59	01/03/2011<br>10:17	1315.2	37.5<br>50
ISO-14_{Total}<br>ISO-14_{Leach}	-0.80	-7.00	25.31	25.00	01/03/2011<br>11:41	03/03/2011<br>13:15	3000.6	50<br>50
ISO-16_{Total}<br>ISO-16_{Leach}	-7.15	-3.25	24.99	25.54	03/03/2011<br>14:17	05/03/2011<br>11:10	2698.2	25<br>25
ISO-19_{Total}<br>ISO-19_{Leach}	-2.96	1.68	25.61	26.25	05/03/2011<br>17:16	07/03/2011<br>19:15	2219.4	25<br>50
ISO-21_{Total}<br>ISO-21_{Leach}	1.81	6.74	26.29	27.80	07/03/2011<br>20:05	09/03/2011<br>19:24	2855.4	50<br>50
ISO-23_{Total}<br>ISO-23_{Leach}	6.84	11.31	27.84	28.86	09/03/2011<br>20:05	11/03/2011<br>19:18	2845.8	50<br>50
ISO-25_{Total}<br>ISO-25_{Leach}	11.45	17.42	28.98	28.38	11/03/2011<br>20:07	14/03/2011<br>11:05	3798.6	50<br>50

[a] Name of sample, subscripts 'Total' and 'Leach' denote subsamples subjected to the total digestion and leaching procedures respectively.
[b] Latitude and longitude of start and end of each aerosol sample collection.
[c] Date (day/month/year) and time (hour:minute) of the start and end of each aerosol collection.
[d] Volume of air sampled during each aerosol collection period.
[e] The proportion of the total exposed filter area taken for the total digestion and leachate subsamples.

Supplementary Table 7; Details for ion exchange chromatography procedure for the separation of Pb from the aerosol total digests and leachates. Procedure adapted from Paul et al.[16].

Resin	100 µl AG1 X8 100 – 200 mesh
Clean resin	3 x 3 ml 0.1 M HNO_3
Equilibrate resin	2 x 0.1 ml 2 M HBr
Load sample	in 3 ml 2 M HBr
Elute matrix	2 x 0.1 ml 2 M HBr
	2 x 0.5 ml 0.5 M HNO_3 – 0.2 M HBr
	0.2 ml 0.5 M HNO_3 – 0.03 M HBr
Elute Pb	3 ml 0.5 M HNO_3 – 0.03 M HBr

Supplementary Table 8; Results of analyses for standard and geological reference materials.

**Sample ID**	**n[a]**	**$^{206}Pb/^{204}Pb$[b]**	**$^{206}Pb/^{207}Pb$[b]**	**$^{206}Pb/^{208}Pb$[b]**
10 ng NIST 981	9	16.9411 ± 19	1.0931 ± 01	2.3695 ± 01
relative 2sd (ppm)[c]		109	56	51
typical within run 2se (ppm)[d]		70	15	15
20 ng NIST 981	6	16.9418 ± 23	1.0931 ± 01	2.3695 ± 02
relative 2sd (ppm) [c]		133	49	82
typical within run 2se (ppm) [d]		40	15	15
Literature				
[17] (TIMS)		16.9405	1.0932	2.3697
[18] (TIMS)		16.9409	1.0933	2.3699
[19] (TIMS)		16.9356	1.0934	2.3694
[20] (MC-ICP-MS)		16.9417	1.0930	2.3694
[21] (MC-ICP-MS)		16.9416	1.0930	2.3694
20 ng BCR-2	9	18.7603 ± 85	1.2006 ± 01	2.4692 ± 03
relative 2sd (ppm) [c]		453	105	131
typical within run 2se (ppm)		90	20	16
Literature				
[21] (MC-ICP-MS)		18.7650	1.2011	2.4804
[22] (TIMS)		18.7500	1.2008	2.4786
[23] (MC-ICP-MS)		18.7570	1.2005	2.4784
[24] (TIMS & MC-ICP-MS)		18.7529	1.2002	2.4783
20 ng BHVO-2	4	18.6398 ± 63	1.1998 ± 02	2.4612 ± 03
relative 2sd (ppm) [c]		336	127	121
typical within run 2se (ppm) [d]		100	25	25
Literature				
[21] (MC-ICP-MS)		18.6490	1.1997	2.4605
[22] (TIMS)		18.6410	1.1997	2.4603
[23] (MC-ICP-MS)		18.6790	1.2003	2.4602
[24] (TIMS & MC-ICP-MS)		18.6474	1.2005	2.4616

[a]Number of replicate analyses conducted.
[b]Mean and absolute uncertainty (2sd) of the replicate analyses.
[c]Relative uncertainty (2sd) of the replicate analyses.
[d]Typical relative internal precision (2se) of a single analysis.

Supplementary Note 1. Literature compilation of Pb isotope data for potential Pb sources to the tropical North Atlantic

The selection criteria for literature data used to assess the isotope composition of anthropogenic Pb transported by easterly winds were as follows: (i) aerosols had to be collected either within the easterly winds (as assessed by air mass back trajectories) in the tropical North Atlantic[1] or within North Africa[2,3], (ii) aerosol results had to be reported for at least the isotopes ^{206}Pb, ^{207}Pb and ^{208}Pb, and (iii) aerosol results had to be produced from dilute acid leaching procedures (typically dilute HBr), rather than from total digestions (featuring concentrated HF) (Supplementary Table 2). The latter was to ensure that the Pb isotope compositions reflected those of anthropogenic emissions, unaffected by mineral dust contributions. The selected data are for aerosols collected during the mid-1900s and 2000s. Recent data is more desirable as the isotope composition of anthropogenic Pb emissions from particular regions can change over decadal time scales, due to changes in the market share of the Pb ores that supply anthropogenic activities and/or changes in anthropogenic activities (e.g. ref[4]). However, the compiled literature data span a similar range to our own leachate results, suggesting a lack of temporal variability in the Pb isotope composition of the emissions for the time span covered by the different sample sets.

For the assessment of the isotope composition of anthropogenic Pb transported by the westerly winds, we use the comprehensive data set of Bollhofer & Rosman[2] for aerosols collected in the eastern US and Central America in the 1990s. These data were acquired on dilute HBr aerosol leachates (Supplementary Table 3). In addition, three North American aerosols collected over the Atlantic in 2011 during the GEOTRACES GA03 section are used for assessment of this endmember[5]. These data were acquired on 'instantaneous' deionized water leaches, following the techniques of Buck et al.[6]. The recent collection dates of these aerosols make them well suited to assess the isotope composition of this endmember Pb source at the time of sample collection for this study, thus they are denoted by their own symbols in Figure 3 (main article). Importantly, the samples north of 20°N in the western tropical Atlantic, evolve towards the composition of these recent North American aerosols in Figure 3 (main article), supporting the interpretation that these surface waters contain contributions of anthropogenic Pb transported by westerly winds.

The Pb isotope composition of the Amazon outflow was assessed using results obtained suspended particles collected within the Amazon River system, as dissolved Pb data are not available[7,8] (Supplementary Table 4).

The Pb isotope composition of North African mineral dust was assessed through (i) data for pre-anthropogenic (Pleistocene) marine sediments in the eastern tropical North Atlantic[9,10], and (ii) data for dust collected within the Bodélé depression (located in Chad)[11] (Supplementary Table 5). The former are interpreted to represent accumulation of North African mineral dust on the ocean floor, while the latter represent dust samples from the most important mineral dust source area in North Africa (and the world)[12]. We note that both data sets display similar Pb isotopic compositions. A single outlier from the data set of Abouchami & Zabel[10] was omitted from the compilation.

Supplementary Note 2. Mass balance estimates for the relative proportions of mineral dust Pb (Pb_{min}) and anthropogenic Pb (Pb_{anth}) in ocean surface waters and aerosol samples

The maximum proportions of Pb_{min} relative to Pb_{anth} in ocean surface waters were estimated using an isotope mass balance approach. The estimates assume that (1) Pb in surface waters consists of a binary mixture of Pb_{min} and Pb_{anth} transported by easterly winds, and that (2) the Pb isotope composition of these two endmembers is represented by the mean of the relevant compiled data sets. For the isotope composition of Pb_{anth} (transported by the easterly winds), the compiled literature data are combined with the leachate data for aerosols collected north of the ITCZ (groups 'North African' and 'Algerian') from this study. The Pb isotope compositions of Pb_{anth} transported by the easterly winds span a relatively large range (Fig. 3; main article), with this variability occurring over periods of several days. This likely reflects the relatively short residence time of atmospheric aerosols (~several days)[13]. Due to the longer residence time of Pb in ocean surface waters (likely on the order of months to several years[14,15]; see discussion in main article), it can be expected that variable inputs of Pb_{anth} will be naturally 'averaged' by mixing. This idea is supported by the smooth and systematic spatial distribution of Pb isotope compositions in the ocean surface (Fig. 2; main article).

Based on the compiled data, we derive that Pb_{anth} (transported by easterly winds) is characterized by $^{206}Pb/^{207}Pb$ = 1.1532 ± 0.0057 and $^{208}Pb/^{207}Pb$ = 2.4304 ± 0.0063 (mean ± 2se, n = 25), whilst Pb_{min} is characterized by $^{206}Pb/^{207}Pb$ = 1.2051 and $^{208}Pb/^{207}Pb$ = 2.4972 (mean, n = 30). Using these endmembers we have constructed a mixing line in $^{206}Pb/^{207}Pb$ versus $^{208}Pb/^{207}Pb$ space (see Fig. 3, main article) based on equation 1:

$$R_{mix} = (R_{min} \text{ x } f_{min}) + (R_{anth} \text{ x } f_{anth}) \quad (1)$$

where R_{mix}, R_{min} and R_{anth} denote the Pb isotope ratios ($^{206}Pb/^{207}Pb$ or $^{208}Pb/^{207}Pb$) of the sample, mineral dust endmember and anthropogenic endmember, respectively. Symbols f_{min} and f_{anth} denote the fraction of sample Pb from the mineral dust and anthropogenic endmembers respectively, with $f_{min} + f_{anth} = 1$. Note that in Figure 3 (main article), f_{min} is expressed as a percentage. Comparison of the Pb isotope compositions of the surface seawater samples with the mixing line indicates maximum Pb_{min} contributions of 30 to 50% (Fig. 3; main article).

A similar isotope mass balance approach was applied to estimate the relative contributions of Pb_{min} to the total Pb budgets of the total aerosol digest samples (eqn. 1). To account for the variability of Pb_{anth} isotope compositions between aerosol samples, the isotopic data for the leachates were assumed to be representative for R_{anth} in the corresponding total digest. As with the surface water mass balance estimates, the mean isotope ratios of the compiled mineral dust literature dataset ($^{206}Pb/^{207}Pb$ = 1.2051 and $^{208}Pb/^{207}Pb$ = 2.4972; Supplementary Table 5), were taken to represent R_{min}. The f_{min} values calculated for each aerosol total digest are expressed as a percentage (Table 2, Fig. 5; main article), and were determined from $^{206}Pb/^{207}Pb$ ratios. However, calculations using $^{208}Pb/^{207}Pb$ yield similar results (Supplementary Figure 4). Uncertainty in these mass balance estimates predominantly arises from the uncertainty in the values of R_{min} for each individual aerosol, and was assessed by propagating the full range of mineral dust isotope compositions from the literature data through the calculation (Supplementary Figure 5).

Supplementary References

[1] Witt, M., Baker, A. R., & Jickells, T. D., Atmospheric trace metals over the Atlantic and South Indian Oceans: Investigation of metal concentrations and lead isotope ratios in coastal and remote marine aerosols, *Atmospheric Environment*, **40**, 5435-5451, (2006)

[2] Bollhofer, A., & Rosman, K. J. R., Isotopic source signatures for atmospheric lead: The Northern Hemisphere, *Geochimica et Cosmochimica Acta*, **65**, 1727-1740, (2001)

[3] Kumar, A., Abouchami, W., Galer, S. J. G., Garrison, V. H., Williams, E., & Andreae, M. O., A radiogenic isotope tracer study of transatlantic dust transport from Africa to the Caribbean, *Atmospheric Environment*, **82**, 130-143, (2014)

[4] Hurst, R. W., Lead isotopes as Age-sensitive Genetic Markers in Hydrocarbons. 3. Leaded Gasoline, 1923-1990 (ALAS Model), *Environmental Geosciences*, **9** (2), (2002).

[5] Noble, A. E et al., Dynamic variability of dissolved Pb and Pb isotope composition from the U.S. North Atlantic GEOTRACES transect, *Deep-Sea Research Part II-Topical Studies in Oceanography*, **116**, 208-225, (2015)

[6] Buck, C. S., Landing, W. M., Resing, J. A. & Lebon, G. T., Aerosol iron and aluminum solubility in the northwest Pacific Ocean: Resulats from the 2002 ICO cruise, *Geochemistry, Geophysics, Geosystems*, **7**, 1524-2027 (2006)

[7] Asmerom, Y., & Jacobsen, S. B., The Pb Isotopic Evolution of the Earth - Inferences from River Water Suspended Loads, *Earth and Planetary Science Letters*, **115** (1-4), 245-256, (1993)

[8] Allègre, C. J., Dupre, B., Negrel, P., & Gaillardet, J., Sr-Nd-Pb isotope systematics in Amazon and Congo River systems: Constraints about erosion processes, *Chemical Geology*, **131** (1-4), 93-112, (1996)

[9] Chow, T. J., & Patterson, C. C., The occurrence and significance of lead isotopes in pelagic sediments, *Geochimica et Cosmochimica Acta*, **26** (2), 263-308, (1962)

[10] Abouchami, W., & Zabel, M., Climate forcing of the Pb isotope record of terrigenous input into the Equatorial Atlantic, *Earth and Planetary Science Letters*, **213** (3-4), 221-234, (2003)

[11] Abouchami, W. et al., Geochemical and isotopic characterization of the Bodele Depression dust source and implications for transatlantic dust transport to the Amazon Basin, *Earth and Planetary Science Letters*, **380**, 112-123, (2013)

[12] Goudie, A. S., & Middleton, N. J., Saharan dust storms: nature and consequences, *Earth-Science Reviews*, **56** (1-4), 179-204, (2001).

[13] Raes, F. et al., Formation and cycling of aerosols in the global troposphere, *Atmospheric Environment*, **34** (25), 4215-4240, (2000).

[14] Bacon, M. P., Spencer, D. W., & Brewer, P. G., ^{210}Pb-^{226}Ra and ^{210}Po-^{210}Pb Disequilibria in Seawater and Suspended Particulate Matter, *Earth and Planetary Science Letters*, **32** (2), 277-296, (1976)

[15] Rigaud, S., Stewart G., Baskaran, M., Marsan, D. & Church, T., ^{210}Po and ^{210}Pb distribution, dissolved-particulate exchange rates, and particulate export along the North Atlantic US GEOTRACES GA03 section, *Deep-Sea Research Part II-Topical Studies in Oceanography*, **116**, 60-78, (2015)

[16] Paul, M., Bridgestock, L., Rehkamper, M., van de Flierdt, T., Weiss, D., High-precision measurements of sweater Pb isotope compositions by double spike thermal ionization mass spectrometry, *Analytica Chimica Acta*, **863**, 59-69 (2015)

[17] Galer, S., & Abouchami, W., Practical Application of lead triple spiking for correction of instrumental mass discrimination, *Mineralogy Magazine*, **62A**, 491-492, (1998)

[18] Thirlwall, M. F., Inter-laboratory and other errors in Pb isotope analyses investigated using a ^{207}Pb-^{204}Pb double spike, *Chemical Geology*, **163** (1-4), 299-322, (2000)

[19] Todt, W., Cliff, R. A., Hanser, A., & Hofmann, A. W., Evaluation of a 202Pb-205Pb double spike for high-precision lead isotope analysis, Paper presented at the Geophysical Monographs Series, American Geophysical Union, Washington, D.C (1996)

[20] Thirlwall, M. F., Multicollector ICP-MS analysis of Pb isotopes using a (207)pb-(204)pb double spike demonstrates up to 400 ppm/amu systematic errors in Tl-normalization, *Chemical Geology*, **184** (3-4), 255-279, (2002)

[21] Baker, J., Peate, D., Waight, T., & Meyzen, C., Pb isotopic analysis of standards and samples using a ^{207}Pb-^{204}Pb double spike and thallium to correct for mass bias with a double-focusing MC-ICP-MS, *Chemical Geology*, **211** (3-4), 275-303, (2004)

[22] Woodhead, J. D., & Hergt, J. M., Pb-isotope analyses of USGS reference materials, *Geostandards Newsletter-the Journal of Geostandards and Geoanalysis*, 24 (1), 33-38, (2000)

[23] Collerson, K. D., Kamber, B. S., & Schoenberg, R., Applications of accurate, high-precision Pb isotope ratio measurement by multi-collector ICP-MS, *Chemical Geology*, **188 (**1-2), 65-83, (2002)

[24] Weis, D. et al., High-precision isotopic characterization of USGS reference materials by TIMS and MC-ICP-MS, *Geochemistry Geophysics Geosystems*, **7**, (2006)

[25] Draxler, R.R. & Rolph, G.D., HYSPLIT (HYbrid Single-Particle Lagrangian Integrated Trajectory) Model access via NOAA ARL READY Website (http://www.arl.noaa.gov/ready/hysplit4.html). NOAA Air Resources Laboratory, Silver Spring, MD (2003)
